# Adaptive estimation of the Gutenberg–Richter *b* value using a state space model and particle filtering

**DOI:** 10.1038/s41598-024-54576-x

**Published:** 2024-03-05

**Authors:** Daichi Iwata, Kazuyoshi Z. Nanjo

**Affiliations:** 1 OPT, Inc., Tokyu Bancho Bldg., 6 Yonbancho, Chiyoda-ku, Tokyo, 102-0081 Japan; 2 Global Center for Asian and Regional Research, University of Shizuoka, 3-6-1, Takajo, Aoi-ku, Shizuoka, 420-0839 Japan; 3 Center for Integrated Research and Education of Natural Hazards, Shizuoka University, 836, Oya, Suruga-ku, Shizuoka, 422-8529 Japan; 4 Institute of Statistical Mathematics, 10-3, Midori-cho, Tachikawa, Tokyo 190-8562 Japan; 5 Japan Agency for Marine-Earth Science and Technology, Yokohama Institute for Earth Sciences, 3173-25 Showa-machi, Kanazawa-ku, Yokohama, Kanagawa 236-0001 Japan

**Keywords:** Health care, Health occupations, Medical research, Rheumatology

## Abstract

Earthquakes follow an exponential distribution referred to as the Gutenberg–Richter law, which is characterized by the *b* value that represents a ratio of the number of large earthquakes to that of small earthquakes. Spatial and temporal variation in the *b* value is important for assessing the probability of a larger earthquake. Conventionally, the *b* value is obtained by a maximum-likelihood estimation based on past earthquakes with a certain sample size. To properly assess the occurrence of earthquakes and understand their dynamics, determining this parameter with a statistically optimal method is important. Here, we discuss a method that uses a state space model and a particle filter, as a framework for time-series data, to estimate temporal variation in the *b* value. We then compared our output with that of a conventional method using data of earthquakes that occurred in Tohoku and Kumamoto regions in Japan. Our results indicate that the proposed method has the advantage of estimating temporal variation of the *b* value and forecasting magnitude. Moreover, our research suggests no heightened probability of a large earthquake in the Tohoku region, in contrast to previous studies. Simultaneously, there is the potential of a large earthquake in the Kumamoto region, emphasizing the need for enhanced monitoring.

## Introduction

The magnitude frequency of earthquakes follows an exponential distribution, and when the magnitude is converted to seismic energy, it follows a power-law distribution, the Gutenberg–Richter (GR) law in which the number of earthquakes with a magnitude over *M*, *n*(*M*), is approximated as follows: $\log {n(M)}=a - b M$ (ref.^1^). The *a* value represents seismic activity or earthquake productivity while the *b* value represents the slope of the exponential or the power-law distribution and indicates a ratio of the number of large earthquakes to that of small earthquakes. Spatial and temporal variations in the *b* value are known to indicate structural heterogeneity, strength, and temperature within the seismicity area^2–4^. Experimental research demonstrated a negative correlation between the *b* value and differential stress in controlled laboratory settings^5^. Furthermore, observational research revealed an inverse association between the *b* value and the slip-deficit rate at plate boundaries^6^. These findings suggest that detailed analysis of the *b* value could allow it to serve as a stress proxy, potentially aiding in the identification of asperities or highly stressed regions at the plate boundary where future large earthquakes are expected to occur. Hence, accurate and real-time estimation is important for assessing the probability of a larger earthquake.

The *b* value of the GR law is conventionally estimated based on the maximum likelihood estimation (see Eq. (1) in Calculating method for the *b* value of the Methods section)^7^. The maximum likelihood method for the *b* value, which requires calculating the mean magnitude of earthquake samples, is controlled by sample size, as a parameter called ‘window width’, which has a temporal meaning in the present study.

In terms of the logarithmic linear fitting of a power-law distribution, the average error of the estimated scaling parameter is reduced to less than 1% once sample size exceeds 50 (ref.^8^), and 50 events were adopted as the minimum number of events for stable *b* value estimation in various studies^4,9,10^. Furthermore, a previous study^11^ illustrated that a more robust estimation of *b* value stability requires the inclusion of a space-time window encompassing 100 earthquakes. Complementing these findings, it has been proposed that a minimum of 200 events is essential to compute the *b* value^10,12^. More recently, a suggested minimum data volume of 300 events was required for accurate *b* value estimation^13^. Although a large window width can provide statistically accurate estimates, it provides a lower time resolution, making it difficult to estimate the detailed temporal properties of the *b* value. Conversely, a smaller window width, while offering higher resolution of time, may lead to less statistically accurate estimates due to a reduced data volume. The optimal window width of the conventional method for estimating the *b* value needs to be set to an appropriate value between a large window width and a small one. Furthermore, seismic activity varies in time, suggesting that the optimal window width also varies in time.

The state space model is an elegant statistical framework for describing time series data, with practical applications in various research fields due to its flexibility for interpreting observed data^14–18^. Simple state space models, such as linear and Gaussian type models, can be estimated efficiently using the Kalman-filter^19^. A nonlinear and non-Gaussian state space model, in which the Kalman-filter might not be effective, can be estimated using a particle filter, also known as the sequential Monte Carlo method. The particle filter approximates the posterior probability density function of the state variables using a set of particles, in which each particle represents a possible state of the system and its weight reflects the likelihood of the observations^14,15,17^. A flexible and widely applicable method that combines a state space model and a particle filter enables real-time estimation that robustly follows unsteady-changing objects such as the *b* value of the GR law. This enables real-time forecasting of seismic activity.

**Figure 1:**
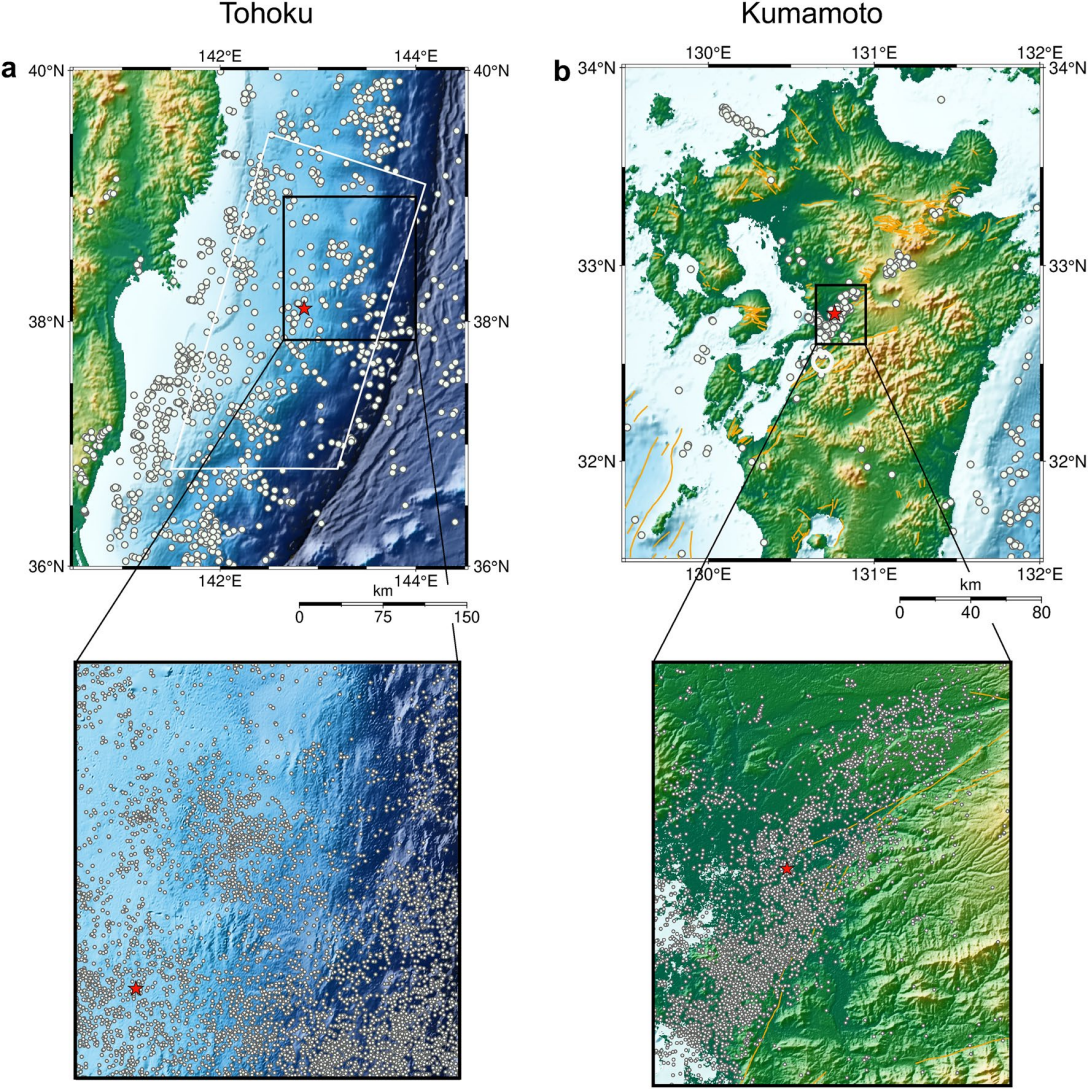
Study regions in (**a**) Tohoku and (**b**) Kumamoto. Earthquakes bounded by black rectangular and white areas in the top panel in **a** and **b** were used to create Fig. 2, and Fig. 4, respectively. Circles in the top panels indicate earthquakes with a magnitude greater than 5.0 (**a**) and 4.0 (**b**). Red stars show the epicenter of the 2011 Tohoku earthquake and 2016 Kumamoto earthquake. Orange lines in **b** indicate active faults. Bottom panels: zoomed areas are displayed with all earthquakes used to estimate the *b* value with a magnitude greater than 2.5 (Tohoku) and 2.0 (Kumamoto). This map was generated using Generic Mapping Tools (version 6.2.0, https://www.generic-mapping-tools.org/).

In this study, we propose such a method to estimate the temporal variation in the *b* value. The parameter, which corresponds to the window width of the conventional method and determines the ability to adapt to variation in the *b* value, is automatically adjusted from the data to the optimum value in our method. To demonstrate our method’s effectiveness for real-time monitoring of the *b* value, we incorporated earthquake data before and after the 2011 Tohoku earthquake (Fig. 1a) and the 2016 Kumamoto earthquake (Fig. 1b). Previous studies suggested a rise in *b* values in these regions following the main shock, but these subsequently decreased to lower levels, implying an increase in probability of a larger earthquake^2,20^. Therefore, we also estimated the *b* value with our proposed method using more recent data, under the same regional and magnitude conditions as previous studies^2,20^, and evaluated whether the *b* value remained low, thus indicating a high probability of a large earthquake. Moreover, we assessed the ability of forecasting magnitude compared to using the conventional approach.

## Results

### Comparison of methods for estimating the *b* value

**Figure 2:**
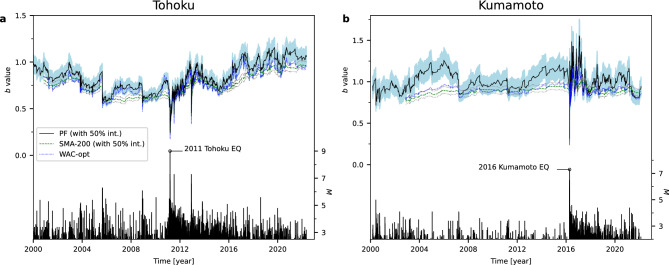
*b* value of the GR law estimated with a particle filter and a conventional method. (**a**) Tohoku and (**b**) Kumamoto cases. **a**, Earthquakes in the black rectangle in Fig. 1**a** were used. **b**, Same as a for Fig. 1**b**: Black line and filled area indicate median and 50% area of the posterior distribution of the *b* value estimated by a particle filter (Model 1: Eqs. (8) and (9) in Methods. The results of Models 2–4 are displayed in Supplementary Fig. S1–S3). Green dashed and blue dotted lines indicate the *b* value estimated by conventional method SMA-200 and the WAC-opt (see Methods for details), respectively. The gray dotted line around SMA-200 indicates the 50% confidence interval of the *b* value computed by bootstrapping^21^. The stem plot in **a** and **b** shows the magnitude of earthquakes. The magnitudes of the Tohoku and Kumamoto earthquakes are highlighted by circles.

Figure 2 shows a comparison of estimated *b* values using a particle filter, the simple moving averages with a fixed length of 200 events (SMA-200), and the weighted average of the conventional method (WAC-opt). The *b* value was determined for every earthquake event. Figure 2a and 2b are results for data of two areas, the 2011 Tohoku earthquake (*M* 9.0; 11 March 2011) and the 2016 Kumamoto earthquake (*M* 7.0; 16 April 2016), respectively. Details of analytical conditions for the data are described in the earthquake catalog section of the Methods. It should be noted that the estimated *b* value at each time point was calculated based exclusively on previous data and was not influenced by subsequent data. In both datasets, before and after the Tohoku earthquake (Fig. 2a) and before and after the Kumamoto earthquake (Fig. 2b), the *b* value estimated by the particle filter reveals more detail of temporal variations than the moving average based on the conventional method. The estimated *b* value for the dataset before and after the 2011 Tohoku earthquake indicates similar fluctuations for the WAC-opt and particle filter methods (Fig. 2a). In contrast, the estimated *b* values for the dataset before and after the Kumamoto earthquake indicates a smaller fluctuation in the WAC-opt compared to the particle filter method (Fig. 2b). The particle filter method enabled us to capture the uncertainty of estimation, and the credible interval of the *b* value’s posterior distribution using Bayesian statistics (filled area in Fig. 2a, b). The procedure for calculating the posterior distribution using the particle filter is presented in the Methods section (see Algorithm for particle filter for details).

The conventional method to estimate the *b* value using a time window (e.g., SMA in Fig. 2) can also be used to evaluate its uncertainty, such as the statistical approach^11^ and the bootstrap method^21^. The methodologies for assessing the uncertainty of the *b* value involve statistical assumptions, statistical independence within a time window, and the same sample weight. It is important to note that the term ‘uncertainty’ in the method of uncertainty estimation and our proposed methods refer to statistically different concepts. The uncertainty that Shi and Bolt referred to^11^ is based on the asymptotic behavior of the variance of a score function, assuming that data within the same time window independently follow an exponential distribution. In contrast, the bootstrap method^21^ does not require parametric assumptions and evaluates the variability in estimates of the *b* value through resampling, assuming data independence within the same distribution. The state space model and particle filter in our method, grounded in Bayesian statistics, differs from these as it provides the posterior distribution of the *b* value. Being based on a time series model, the estimated uncertainty indicates uncertainty exclusively at time *t*.

Moreover, more detailed characteristics of the temporal variation of the *b* value were clarified by applying the particle filter method, resulting in a better forecast of the magnitude. Before the 2011 Tohoku earthquake, the *b* value had been decreasing over time. This long-term decreasing trend of the *b* value in Tohoku is consistent with that observed in previous studies based on the conventional method^2,22^. In addition to these characteristics, the more detailed variation of *b* estimated in this study suggests that the decreasing trend was not a monotonic decrease, but instead a stepwise decreasing variation associated with the occurrence of earthquakes with a magnitude of about 4 to 6. Moreover, the trend did not recover to the original level afterwards, i.e., at the end of 2008 and before the 2011 Tohoku earthquake. After the 2011 Tohoku earthquake, an opposite pattern was observed, namely an increase in the *b* value after the main shock, with a recovery to its original level about 2 years later.

Variation of the *b* value before the 2016 Kumamoto earthquake indicates little change, consistent with the findings of a previous study^23^. After the 2016 Kumamoto earthquake, the *b* value increased to about 1.0, finally equilibrating at around 0.8, which is the level between 2000 and 2016. Focusing on more detailed patterns, a tendency to oscillate can be seen between 0.6 and 0.8, from a sharp decrease to a slight increase after earthquakes, when dividing the time line into three periods: 2000–2006, 2006–2011, and 2011–2016.

### Evaluating performance of the estimated *b* value for forecasting magnitude

The evaluation of estimated *b* values raises a fundamental question: How can these estimations be accurately assessed? It is important to note that *b* values are not directly measurable; instead, they are inferred from observed magnitudes. One approach to evaluate the precision of *b* value estimations involves generating artificial data, which includes *b* values and corresponding magnitudes, and then comparing the estimated results with the true values within this dataset^24^. We utilized this approach with artificial data, which implied the effectiveness of our proposed method (see Supplementary Figs. S6, S12 and S13 and Supplementary Materials S2 for details). However, it remains unclear whether these results are equally valid when applied to actual observational data. Therefore, instead of evaluating the accuracy of the *b* value estimates, we assessed how close the magnitudes predicted based on these estimated *b* values were.

**Figure 3:**
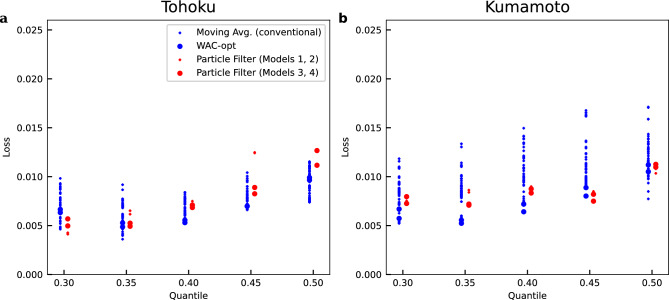
Evaluating the performance of forecasting magnitude. (**a**) Tohoku and (**b**) Kumamoto cases. The abscissa represents the level of the quantile of predictive distribution of magnitude for evaluating performance. Marks indicate how close, shown as a value in the ordinate, the percentage exceeded the quantile (abscissa) of the predictive distribution of magnitude, calculated based on the estimated *b* value, is to the theoretical value. The *b* values are estimated with conventional methods and particle filter method with an earthquake catalog of magnitude over the time-varying magnitude completeness $M_{\textrm{c}}$ (Supplementary Fig. S11). The output of the particle filter is represented by red markers in each quantile *q*, while the output of the moving average based on the conventional method, and comprising 21 (3 kind of moving average methods times 7 kind of window length) points, is shown by blue markers. To avoid overlapping of marks, and to facilitate visualization, -0.005 or +0.005 were added to the value along the abscissa for blue and red marks, respectively. Models 3 and 4 with the state space model and the particle filter method tuned the hyper-parameter automatically unlike Models 1 and 2, which were manually tuned. In this context, ‘quantile’ refers to the quantile of the predictive distribution of magnitude. The shape of this predictive distribution of magnitudes varies depending on the *b* value, which varies with time and location. Consequently, as the quantile of the predictive distribution of magnitudes varies with time and location, the corresponding magnitude levels also change. The results of a similar analysis performed on data above magnitude completeness $M_{\textrm{c}}$, which was set commonly for the entire dataset, are shown in Supplementary Fig. S10.

Figure 3 shows the ability of earthquake magnitude forecasting based on the estimated *b* values using the particle filter and conventional methods. The performance of magnitude forecasting was evaluated by comparing loss (Fig. 3), which represents the difference between the number of events that exceeded the *q*-th quantile of predictive distribution of magnitude, meaning predictive distribution calculated based on Bayesian statistics, and the expected number of such events, as described in the Method section and Supplementary Fig. S4.

For the dataset of Tohoku earthquakes, the results of the particle filter (red marks in Fig. 3a) at quantiles of 0.3 and 0.35 tend to indicate less loss than the conventional method (blue marks in Fig. 3a). For the dataset of Kumamoto earthquakes, the results of the particle filter (red marks in Fig. 3b) at each quantile tend to indicate less loss than the conventional method (blue marks in Fig. 3b). The loss of the conventional method strongly depends on the window width and indicates a large deviation among the results at each *q*-th quantile. On the other hand, the loss of the particle filter-based method indicates a similar value at each *q*-th quantile. Therefore, the particle filter method indicates a better forecast of magnitude than the conventional method.

The magnitude completeness $M_{\textrm{c}}$ level has a critical impact on *b* value estimation, as highlighted for instance by a study by Tormann et al.^2^. Hence, to evaluate the influence of $M_{\textrm{c}}$ in estimating the *b* value, instead of calculating $M_{\textrm{c}}$ for different periods to estimate the *b* value, we fixed $M_{\textrm{c}}$ to a single value for the entire dataset and conducted a similar analysis (Supplementary Fig. S10). The results of this analysis particularly show that in the analysis of the dataset from the Tohoku datasets, the loss using traditional methods is significantly high (Supplementary Fig. S10) compared to the results in Fig. 3a. For both datasets, the results of the analysis in which $M_{\textrm{c}}$ was fixed to a single value indicate less loss of the proposed method than that of the conventional method. These findings suggest that the proposed method might estimate the *b* value more robustly and with less influence by the accuracy of the $M_{\textrm{c}}$ setting than the conventional method.

**Figure 4:**
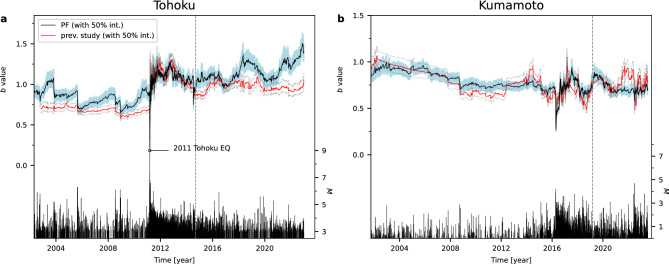
*b* value of the GR law estimated with a particle filter and conventional method using earthquake data extracted under the same conditions as previous studies^2,20^. (**a**) Tohoku and (**b**) Kumamoto cases. Black line and filled area indicate median and 50% area of posterior distribution of the *b* value estimated by a particle filter (Model 4: Eqs. (11) and (10) in Methods). Red dashed lines in (**a**) and (**b**) indicate the *b* value estimated by applying a conventional method SMA of window width 250 and 100, respectively, to data greater than magnitude completeness $M_{\textrm{c}}$ calculated using the EMR method^7^ within the window width. These results are based on the calculation conditions for the *b* value of previous studies (Fig. 3a in ref.^2^, Fig. 3c in ref.^20^, respectively). The gray dotted line around previous study estimates (red dashed line) indicates the 50% confidence interval of the *b* value computed by bootstrapping^21^. The stem plot in each panels shows the magnitude of earthquakes. The gray dashed vertical lines indicates the end date of data analyzed in previous studies^2,20^.

## Discussion

The conventional method of maximum likelihood estimation is limited to a constant *b* value and does not consider its time-series structure. In contrast, the method based on the state space model and particle filtering assumes the time-series structure and can adapt to temporal variation of the *b* value. Supplementary Fig. S5 illustrates the optimal window width for the conventional moving average method based on WAC-opt. The graph indicates that the appropriate window width for the conventional moving average method dynamically varies throughout the observation period (e.g., 2008–2012 in the Tohoku case Supplementary Fig. S5a, and 2004–2008 in the Kumamoto case Supplementary Fig. S5b). In the method of the state space model using the particle filter, the system equation represents the dynamics of the *b* value. The parameter $\sigma _{\log b}$ in the system equation controls the degree of variation of the *b* value (see description of Models 1–4 in the Method for details). Adjusting $\sigma _{\log b}$ to an appropriate value for each observation enables the model to adaptively follow variations in the *b* value. Specifically, incorporating a truncated normal distribution into the dynamics of $\sigma _{\log b}$ in Models 3 and 4 allows adaptation to those changes in the *b* value. Hence, the particle filter estimation provides the temporal variation of the *b* value more adaptively and more accurately, relative to the conventional method for estimating the *b* value.

Figure 4 shows *b* value estimation using earthquake data with the same regions and magnitudes as those employed in previous studies^2,20^. This analysis also incorporated data of time periods subsequent to those examined in those studies. In the Tohoku region, it was reported that the *b* value increased after the 2011 Tohoku earthquake, and then showed a gradual declining trend until around 2015^2^. However, our analysis shows that the *b* value displays an upward trend from around 2015 until 2022, varying at around a value of 1.2 and 1.4 at the end of the data series (Fig. 4a). The latest level of the *b* value suggests that the increase in probability of a large earthquake, which was a concern in a previous study^2^, might not be high. In the Kumamoto region, a decrease in the *b* value at around the Hinagu faults zone (white circle in Fig. 1b) from immediately after the Kumamoto earthquake until 2019 was indicated in a previous study^20^. The result of our analysis indicates that the *b* value has continued to remain at a relatively low level since 2019 (Fig. 4b). This result lends support to the possibility that an increase in probability of a large earthquake in the Hinagu faults zone, which was a concern in a prior study^20^, might still exist. This suggests the need for enhanced monitoring in this area.

With conventional methods, the estimated *b* values and the predicted distribution of magnitudes could be significantly different if the underlying process changes before the next earthquake occurs. This is because traditional methods focus only on what happened within a past window of samples, without any estimation of how *b* values could dynamically change, assuming that the current state of *b* values will persist until the next earthquake. In contrast, our method based on the state space model assumes that *b* values change according to dynamics, as represented in the model (e.g., Eqs. (8) and (11) in Methods). This allows us not only to update estimates from past time $t-1$ to the present time *t*, but also to predict how they will change for the future time $t+1$ based on current estimates. Our study aims to contribute to estimating *b* values and assessing the probability of a large earthquake. To achieve this, it is necessary to consider the possibility of changes in the underlying state until the next earthquake, which makes our proposed method, which employs a state space model and a particle filter, more fitting for the problem than traditional moving average methods or their weighted average.

To facilitate a comparison of methods for estimating the *b* value that approximates real-world conditions, we primarily utilized actual seismic data for validation. However, further validation using synthetic data, as shown in Supplementary Fig. S6 and Supplementary Materials S2, is also considered valuable. Apart from the stepwise varying *b* value demonstrated in our research, several validation patterns can be envisioned, such as those with a gradually changing true *b* value or an oscillating true *b* value. It is believed that conducting these validations will enable us to understand how each method can adaptively estimate the *b* value in response to different types of data. This knowledge is likely to be beneficial when applied to seismic activities that vary in characteristics based on location and period.

In this study, we utilized a particle filter to estimate four models, all of which demonstrated better forecasting of magnitude than a conventional method. However, modeling more detailed variation in the *b* value could potentially improve the ability of forecasting magnitude. In addition, developing a time-series model that does not assume the GR law locally and in the short term, but holds the GR law globally, could allow us to uncover the physical background of the temporal variation in the *b* value. The proposed method is expected to enhance real-time estimation and enables the immediate evaluation and long-term forecasting of probability for a large earthquake through further improvements to these models. Thus, as was shown in our study, applying this approach to various time-series data in the field of earth science has the potential to monitor and mitigate the risk of disasters, contributing to the betterment of our society.

## Methods

### Earthquake catalog

The earthquake dataset used in this study was obtained from the earthquake catalog published by the Japan Meteorological Agency (JMA). To show the applicability of our proposed method for estimating the *b* value, we analyzed earthquake sequences in the Tohoku and Kumamoto regions, observed between 2000 and 2022. Seismicity in the Tohoku region, situated along a plate boundary, is characterized by high seismic activity and large-scale earthquakes, such as the 2011 Tohoku earthquake. On the other hand, the Kumamoto region is characterized by inland seismic activity and a lower frequency of earthquakes. For the analysis of the Tohoku region (Fig. 2a), data of *M* $\ge$ 2.5 and a depth $\le$ 60 km in the region, shown as a black square in Fig. 1a, were used. For the analysis of the Kumamoto region (Fig. 2b), data of *M* $\ge$ 2.0 and a depth $\le$ 25 km in the region, shown as a black square in Fig. 1b, were used. The analyzed region for both datasets was set to that employed in previous studies^22,23^. To estimate the *b* value under the same conditions as previous studies^2,20^, we additionally estimated the *b* value using data of *M* $\ge$ 3.0 in the region shown as a white rectangle in Fig. 1a for the Tohoku region, and data of *M* $\ge$ 0.0 in the region shown as a white circle in Fig. 1b for the Kumamoto region.

### Method for calculating the *b* value

#### Conventional method

The *b* value, conventionally estimated by a maximum-likelihood estimation method, was calculated according to the following formula:

1 $$\begin{aligned} b = \frac{\log _{10}e}{{\bar{M}} - (M_c-\frac{1}{2}M_{bin})} \end{aligned}$$

where ${\bar{M}}$ is the mean magnitude of the dataset and $M_{bin}$ and $M_c$ are the bin width of the catalog and complete magnitude, respectively^1,7,25,26^. In this study, $M_{bin}$ was 0.1. For the result of Fig. 3, $M_{c}$ was set to each divided period (Tohoku: $M_{c} = 2.5$ before 11 March 2011, $M_{c} = 3.5$ between 26 March 2011 and 10 May 2011, $M_{c} = 3.0$ after 10 May 2011, Kumamoto: $M_{c} = 2.0$ before 14 April 2016 and after 20 April 2016, $M_{c} = 2.3$ between 14 April 2016 and 20 April 2016) based on the time series (Supplementary Fig. S11) calculated using Entire-Magnitude Range method^7^. For the result of additional analysis of Supplementary Fig. S10, $M_{c}$ was set to each dataset: $M_c=2.5$ for Tohoku and $M_c=2.0$ for Kumamoto. In order to calculate the time series of the *b* value, the mean magnitude ${\bar{M}}$ in Eq. (1) at time *t* needed to be calculated using moving average $\bar{M_t}$. In this study, three types of moving average—simple, weighted, and exponential smoothing—were used, and analyzed with various window widths of 50, 75, 100, 125, 150, 175, and 200 events. The following is a summary of the methods to calculate each type of moving average.

- Simple moving average (SMA)
  2 $$\begin{aligned} \bar{M_t} = \frac{M_t + M_{t-1} + \cdots + M_{t-s+1}}{s} \end{aligned}$$
  where $M_t$ and $\bar{M_t}$ are observed magnitude and mean value of the magnitude, respectively. The parameter *s* is the window width controlling sample size.
- Exponential moving average (EMA)
  3 $$\begin{aligned} \bar{M_t} = \eta M_t + (1-\eta ){\bar{M}}_{t-1} \end{aligned}$$
  where the value of $\eta = \frac{2}{s+1}$ is according to the Python package^27^.
- Weighted moving average (WMA)
  4 $$\begin{aligned} \bar{M_t} = \frac{sM_t + (s-1)M_{t-1} + \cdots + M_{t-s+1}}{s + (s-1) + \cdots + 1} \end{aligned}$$

In this study, the method combining the moving average type (MA) and window width *s* was denoted as MA-*s*, e.g., SMA-100 for the combination of simple moving average and a window width of 100. The moving average kernels are displayed in Supplementary Fig.  S7.

#### Weighted average of conventional forecasting

The estimation with conventional methods involves the uncertainty of the appropriate window width setting. To address this issue, we introduced a weighted average of the conventional method, WAC-opt (a special case of Bayesian predictive likelihood model averaging method^28,29^) that synthesizes results calculated with different window widths. The *b* value determined by this technique was computed from the following equation:

5 $$\begin{aligned} {\hat{b}}_t = \sum _{k=1}^K w_{t,k} b_{t,k} \end{aligned}$$

where $b_{t,k}$ denotes the *k*-th estimated *b* value among the *K* conventional estimates (number of moving average methods times the number of possible window lengths, here $3\times 7 = 21$), and $w_{t,k}$ represents the weight of the *k*-th method. To calculate $w_{t,k}$, we first obtained the *b* value for each conventional estimate. Next, the weighted average of those *b* values was computed using Eq. (5). The weights in Eq. (5) were determined by:

6 $$\begin{aligned} w_{t,k} = \frac{\exp (-\gamma ({\hat{l}}_{max}-{\hat{l}}_{t,k}))}{\sum _{i=1}^K{\exp (-\gamma ({\hat{l}}_{max}-{\hat{l}}_{t,i}))}} \end{aligned}$$

where the log-likelihood of the *k*-th model is indicated by $l_{t,k}$, and $l_{max}$ represents the largest of $l_{t,1}$ to $l_{t,K}$. Then, the average of the log-likelihood related to the past *N* events is represented by ${\hat{l}}_{t,k}$. The parameter $\gamma$ controls the influence of the difference in log-likelihood between models, but it is difficult to know the appropriate value in advance. The objective of this study is to demonstrate the methodology of combining a state space model and particle filter against estimates derived from conventional methods and their weighted averages. Consequently, the gamma value for the weighted average method was determined after conducting several trials. In this study, $\gamma$ was set to 100. To calculate the log-likelihood $l_{t,k}$, the GR law for magnitude was assumed.

#### State space model for estimating the *b* value

The state space model, a framework for analyzing time-series data, offers flexibility for interpreting observed data and has practical applications in various research fields^14–18^. The observed numerical values were denoted by *y*(*t*) and the internal state of the observed system was denoted by ${\varvec{x}}(t)$. Then, the state space model assumes that *y*(*t*) is observed according to the following equations^30^:

7 $$\begin{aligned} \begin{aligned} {\varvec{x}}(t)&= f({\varvec{x}}(t-1), \eta (t)) \\ y(t)&= h({\varvec{x}}(t), v(t)) \end{aligned} \end{aligned}$$

where, $\eta (t)$ and *v*(*t*) represent noise terms, and the functions $f(\cdot )$ and $h(\cdot )$ are generally nonlinear. The first and second equation are referred to as the system equation and observation equation, respectively. This estimation based on the nonlinear state space model is efficiently performed by using a particle filter^14,15^. Using a particle filter, it is possible to estimate the state ${\varvec{x}}(t)$ based on the observed values ${y(1), \cdots , y(t)}$ obtained until time *t*. A detailed algorithm of the particle filter is presented in the next section. The value estimated using the particle filter at a certain time is based on the data from only before that time, and does not include information after that time. Since the functions $f(\cdot )$ and $h(\cdot )$ in the state space model can be configured flexibly, this allows the model’s estimated values to adapt dynamically to variations in the observational target, such as the time-varying *b* value in the GR law. In this study, we built four models represented by pairs of systems and observation equations, as follows:

- Model 1
  - System equation:
    8 $$\begin{aligned} \log b(t) = \log b(t-1) + \epsilon \end{aligned}$$
    where, the variable $\epsilon$ is a normally distributed random variable with a mean of 0 and a standard deviation of $\sigma _{\log b}$, $\epsilon \sim Normal(0, \sigma _{\log b})$
  - Observation equation: Exponential distribution
    9 $$\begin{aligned} p(M(t)) = \beta (t) \exp (-\beta (t)(M(t)-M_0)) \end{aligned}$$
    $M_0$ represents the lower bound of the magnitude of the earthquake under consideration. There is a relationship between $\beta (t)$ and *b*(*t*) given by $\beta (t) = b(t) \log (10)$.
- Model 2
  - System equation: same as Eq. (8)
  - Observation equation: Exponential with upper bound $M_L$
    10 $$\begin{aligned} p(M(t)) = \frac{\beta (t) \exp (-\beta (t)(M(t)-M_0))}{1 - \exp (-\beta (t)(M_L-M_0))} \end{aligned}$$
    In this study, the $M_L$ value for analysis of the Tohoku region data was set at 9.0, while that for the Kumamoto region data was set at 8.0.
- Model 3
  - System equation:
    11 $$\begin{aligned} \begin{aligned} \log \sigma _{\log b}(t)&= \log \sigma _{\log b}(t-1) + \epsilon _1 \\ \log b(t)&= \log b(t-1) + \epsilon _2 \end{aligned} \end{aligned}$$
    where the variable $\epsilon _1$ is a truncated normally distributed random variable assuming values from -10 to -3 with a mean of 0 and a standard deviation of 0.5, $\epsilon _1 \sim TruncatedNormal(lower=-10, upper=-3, 0, 0.5)$. $\epsilon _2 \sim Normal(0, \sigma _{\log b}(t))$
  - Observation equation: same as Eq. (9)
- Model 4
  - System equation: same as Eq. (11)
  - Observation equation: same as Eq. (10)

In Eq. (8) of Models 1 and 2, $\log {b(t)}$ is a state variable and $\sigma _{\log {b}}$ is a hyperparameter, which is predetermined to maximize likelihood. On the other hand, in Eq. (11) of Models 3 and 4, both $\log {b(t)}$ and $\log {\sigma _{\log {b}}(t)}$ are considered state variables. Unlike Models 1 and 2, here it is assumed that they vary over time and are estimated sequentially from the data without being predetermined. The result of *b* value estimates for artificial data using Model 4 is presented in Supplementary Fig. S6.

The equations in the state space model, as specified in Eqs. (8)–(11), are focused on describing the temporal variations of the *b* value. Models 1 through 4 represent incremental modifications in conditions. The state Eq. (8) in Model 1 represents the gradual temporal variation of the *b* value using a random walk model. In this model, the extent of time variation is constant and denoted as $\sigma _{\log b}$. The observation Eq. (9) in Model 1 denotes that the observed earthquake magnitudes follow the standard GR law, assuming a time-varying *b* value. Model 2 differs from Model 1 in its observation Eq. (10), representing adherence to a truncated GR law. Model 3, unlike Models 1 and 2, allows for the time variation width $\sigma _{\log b}$ of the *b* value to also change over time in Eq. (11). This is to model periods of more rapid or slower changes in the *b* value. The observation equation in Model 3 remains the same as in Model 1, adhering to the standard GR law. Model 4 combines elements from Models 1 through 3. This study presents an introductory exploration into the application of state space models and particle filters to estimate the *b* value, indicating that these models may not fully accurately represent changes in the *b* value and suggesting the need for further model refinement.

#### Algorithm for the particle filter

Particle filtering is a sequential Monte Carlo method used for estimating the state of a dynamic system given a sequence of observations^14–16^. The state variable was denoted as $x_t$ and the observation as $y_t$, where *t* is the time index. The objective was to estimate the posterior distribution $p(x_t|y_{1:t})$ given a set of observations $y_{1:t}$ = $\lbrace y_1, y_2, \cdots , y_t\rbrace$.

The algorithm proceeds as follows:

1. *Initialization* At time $t=0$, generate *N* particles (samples) $x_0^{(i)}$ from the initial state distribution $p(x_0)$. Assign equal weights $w_0^{(i)} = \frac{1}{N}$ to each particle $i=1, 2, \cdots , N$.
  For $$t=1, 2, \cdots , T$$:
  - *Propagation (prediction)* For each particle $i=1, \cdots , N$, draw a new state $x_t^{(i)}$ from the state transition distribution $p(x_t|x_{t-1}^{(i)})$ representing the system dynamics.
  - *Update (correction)* For each particle $i=1, 2, \cdots , N$, compute the importance weight $w_t^{(i)}$ as the likelihood of the observation $y_t$ given the current state $x_t^{(i)}$:
    12 $$\begin{aligned} w_t^{(i)} = p(y_t|x_t^{(i)}) \end{aligned}$$
  - *Normalize the weights such that their sum equals 1*
    13 $$\begin{aligned} w_t^{(i)} \leftarrow \frac{w_t^{(i)}}{W_t} \end{aligned}$$
    for $$i=1, 2, \cdots , N$$, where $$W_t = \sum _i w_t^{(i)}$$.
  - *Resampling* Resample *N* particles from the current set with replacement extraction, with probabilities proportional to the normalized weights $w_t^{(i)}$. This step helps to eliminate particles with low weights and duplicate those with high weights, resulting in a set of particles that better represent the true posterior distribution.

The state transition distribution $p(x_t|x_{t-1}^{(i)})$ is represented by system equations, which in our case are Eqs. (8) and (11). For instance, a transformation of Eq. (8) as noted below in Eq. (14) reveals how it can be considered as a state transition distribution.

14 $$\begin{aligned} \begin{aligned} \log b(t)&= \log b(t-1) + \epsilon , \textrm{where}\quad \epsilon \sim Normal(0, \sigma _{\log b}) \\ \log b(t)&\sim Normal(\log b(t-1), \sigma _{\log b}) \end{aligned} \end{aligned}$$

In the current problem setting, the state variable $x_t$ is represented by $\log {b(t)}$ and $\log \sigma _{\log b}(t)$, as defined in the system Eqs. (8) and (11) of the state space model. The observed values $y_t$ correspond to the magnitudes $M_t$ of the earthquakes. Thus, determining the posterior distribution $p(x_t|y_{1:t})$ of the state implies estimating the state of the *b* value according to the GR law from the magnitudes of earthquakes, which includes accounting for the distribution of its uncertainties. The posterior distribution $p(x_t|y_{1:t})$ can be approximated by the set of particles ${x_t^{(i)}}$ as $p(x_t | y_{1:t}) \simeq \frac{1}{N}\sum _{i=1}^N \delta (x_t - x_t^{(i)})$, where $\delta (x)$ is the delta function $\delta (x) = \infty$ (where $x=0$), otherwise $\delta (x) = 0$ and $\int _{-\infty }^{\infty } \delta (x) dx = 1$. The number of particles *N* was set to $10^5$ in this study. In the context of applying the state space model and particle filter to the present study to estimate temporal variation of the *b* value, the initial distribution $p(x_0)$ refers to the initial distributions of the state variables *b*(0) and $\sigma _{\log b}(t)$. Ideally, these distributions should be based on physical laws. However, in cases like ours where direct observation is difficult, the distribution is set to cover a wide range of values. In this study, we set the initial distribution for $\log {b(0)}$ in Eq. (8) as a normal distribution, $\log {b(0)} \sim Normal(0, \log {10})$, and for $\log {\sigma _{\log {b}}(0)}$ in the first part of Eq. (11) as a uniform distribution, $\log {\sigma _{\log {b}}(0)} \sim Uniform(e^{-10}, e^{-3})$.

### Evaluation method for magnitude forecasting

To compare the estimation of the *b* value from the conventional moving average and from the particle filter, the accuracy of the estimation should be evaluated. The accuracy is the deviation of the estimated *b* value from the true *b* value. However, the true *b* value cannot be observed. Thus, we derived the predictive distribution of magnitude using the *b* values from each method and evaluated their performance of forecasting magnitude using the following procedure (Supplementary Fig. S4). First, we obtained the *q*-th percentile of predictive distribution of magnitude, and then determined whether the magnitude of earthquakes occurring subsequently exceeded the *q*-th percentile or not. If the estimation method adaptively captured the temporal characteristics of the *b* value associated with the time-varying physical environment and accurately predicted them, we expected the probability that the actual magnitude exceeded the *q*-th percentile to be close to *q*. Therefore, the ability of magnitude forecasting was evaluated by following a loss measurement, as the Kolmogorov-Smirnov distance^31^. A smaller loss value indicates better forecasting of magnitude.

15 $$\begin{aligned} loss = \underset{n}{\textrm{max}}\biggl |\frac{N_{exc.}(n) - nq}{N}\biggr | \end{aligned}$$

In this context, $N_{exc.}(n)$ denotes the number of times the cumulative count of events has surpassed the *q*-th percentile of the predictive distribution of magnitude by the time of the *n*-th earthquake. *N* is the total number of events. The notation $\underset{n}{\textrm{max}}|X|$ is used to represent the maximum absolute value of *X* with respect to various values of *n*. The actual values of $N_{exc.}(n)$ obtained in this study are displayed in Supplementary Figs. S8 and S9. The quantiles, which depend on the predictive distribution of magnitudes, were calculated from the *b* values that vary over time. Consequently, the level of magnitude associated with the quantile also changes with the temporal variations in *b* value.

### Supplementary Information

Supplementary Information.

## Data Availability

The datasets that support this study are available from the corresponding author and can be accessed upon reasonable request. The source code is available at the GitHub repository (https://github.com/D-I-29/gr-b-pf) (see Supplementary Material 1 for details).
