# Supplementary material for: Adaptive estimation of the Gutenberg–Richter b value using a state space model and particle filtering
Source: Sci Rep. 2024 Mar 5;14:4630. doi: 10.1038/s41598-024-54576-x (PMC10915173; doi:10.1038/s41598-024-54576-x)
Supplement: Supplementary file 1 — Supplementary Information. [file 41598_2024_54576_MOESM1_ESM.pdf]

Supplementary Materials for "Adaptive estimation of the Gutenberg-Richter b value using a state space model and particle filtering"

Daichi Iwata[1,*] **and Kazuyoshi Z. Nanjo**[2,3,4,5]

[1]OPT, Inc., Tokyu Bancho Bldg., 6 Yonbancho, Chiyoda-ku, Tokyo 102-0081, Japan
[2]Global Center for Asian and Regional Research, University of Shizuoka, 3-6-1, Takajo, Aoi-ku, Shizuoka 420-0839, Japan
[3]Center for Integrated Research and Education of Natural Hazards, Shizuoka University, 836, Oya, Suruga-ku, Shizuoka 422-8529, Japan
[4]Institute of Statistical Mathematics, 10-3, Midori-cho, Tachikawa, Tokyo 190-8562, Japan
[5]Japan Agency for Marine-Earth Science and Technology, Yokohama Institute for Earth Sciences, 3173-25 Showa-machi, Kanazawa-ward, Yokohama City, Kanagawa, 236-0001, Japan
[*]dai.iwata.r@gmail.com

Supplementary Figure S1

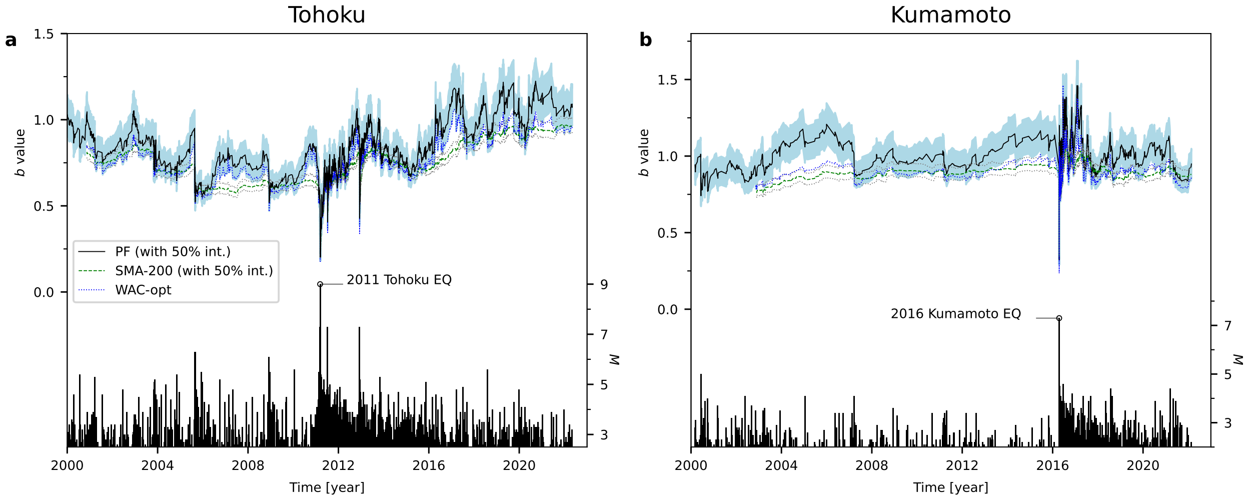

Supplementary Figure 1. The same kind of plots as Fig. 2 in the main text. Black line and filled area indicate median and 50% interval of the posterior distribution of the b value estimated by a particle filter (**model 2**), respectively.

Supplementary Figure S2

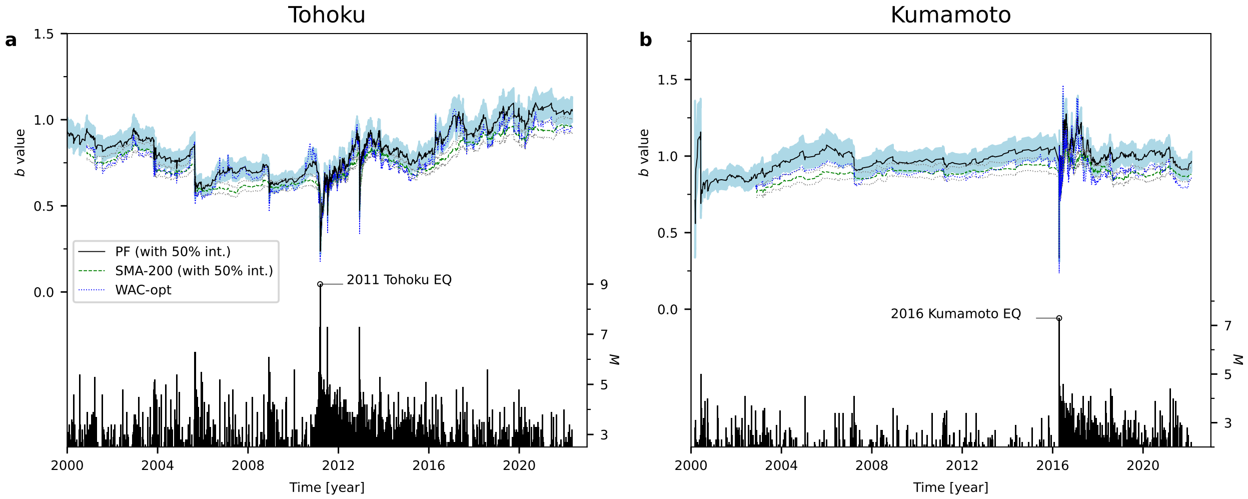

Supplementary Figure 2. The same kind of plots as Fig. 2 in the main text. Black line and filled area indicate median and 50% interval of the posterior distribution of the b value estimated by a particle filter (**model 3**), respectively.

Supplementary Figure S3

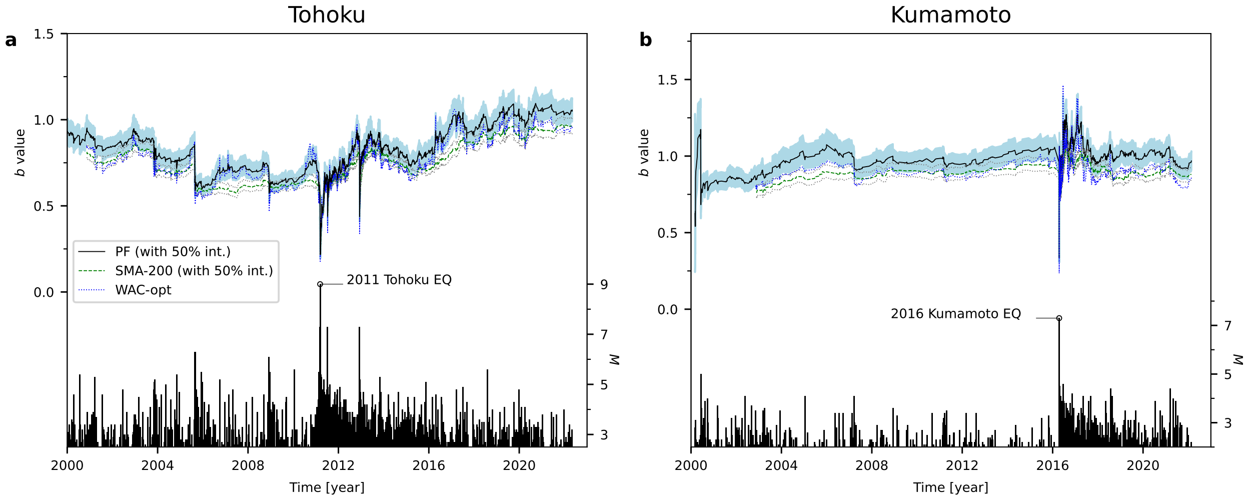

Supplementary Figure 3. The same kind of plots as Fig. 2 in the main text. Black line and filled area indicate median and 50% interval of the posterior distribution of the *b* value estimated by a particle filter (**model 4**), respectively.

Supplementary Figure S4

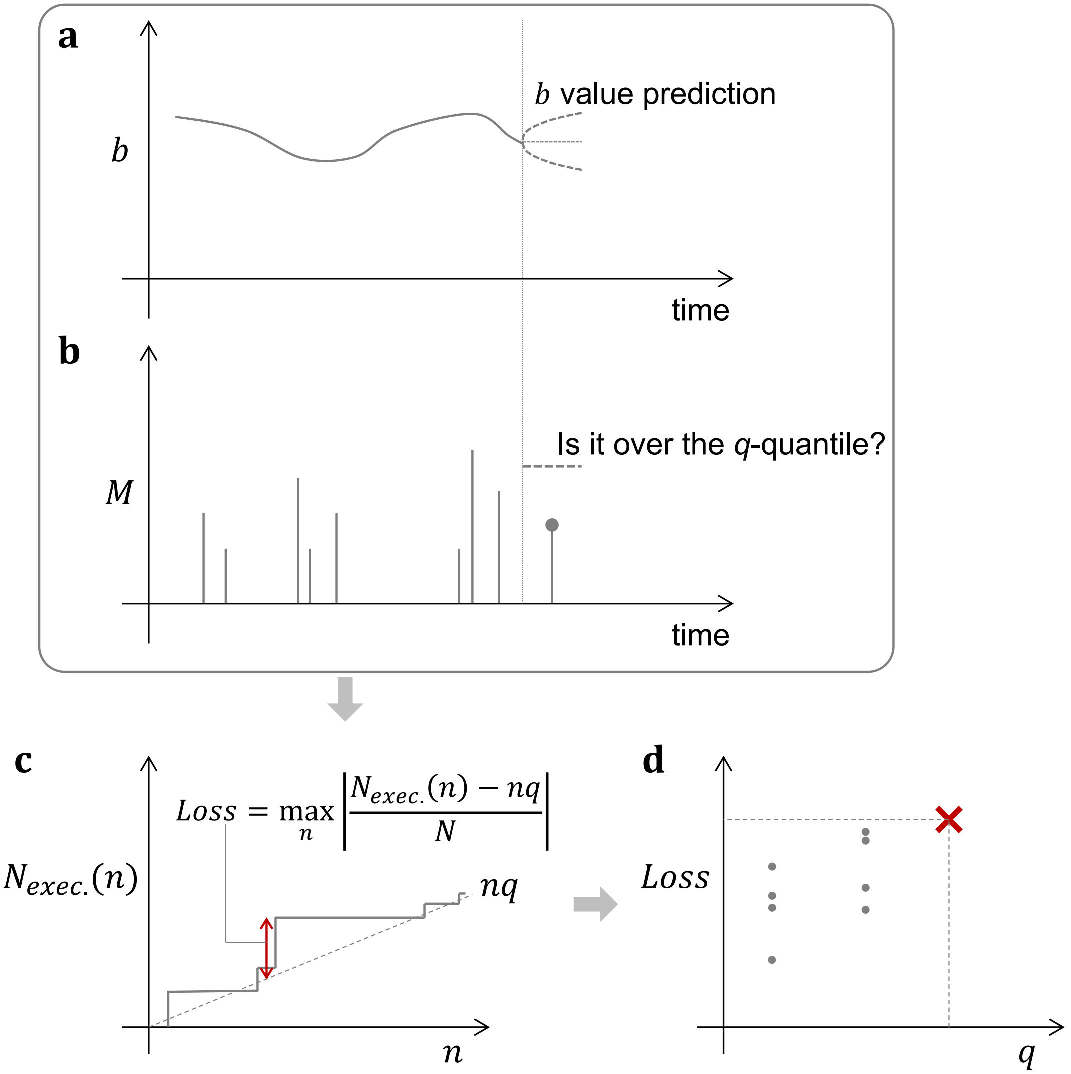

Supplementary Figure 4. The procedure for evaluating a method for predicting the distribution of magnitude is as follows: (**a**) infer the b value one period ahead and calculate the q-th quantile of the predictive magnitude. (**b**) Determine where an actual magnitude exceeds the q-th quantile or not and count the number of events exceeding the threshold. $N_{exec.}(n)$ represents the cumulative number of events until the n-th event. (**c**) Calculate the maximum distance between the two lines $N_{exec.}(n)$ and the expected value nq. This maximum distance is denoted as $Loss$. (**d**) Add a point at $(q, Loss)$.

Supplementary Figure S5

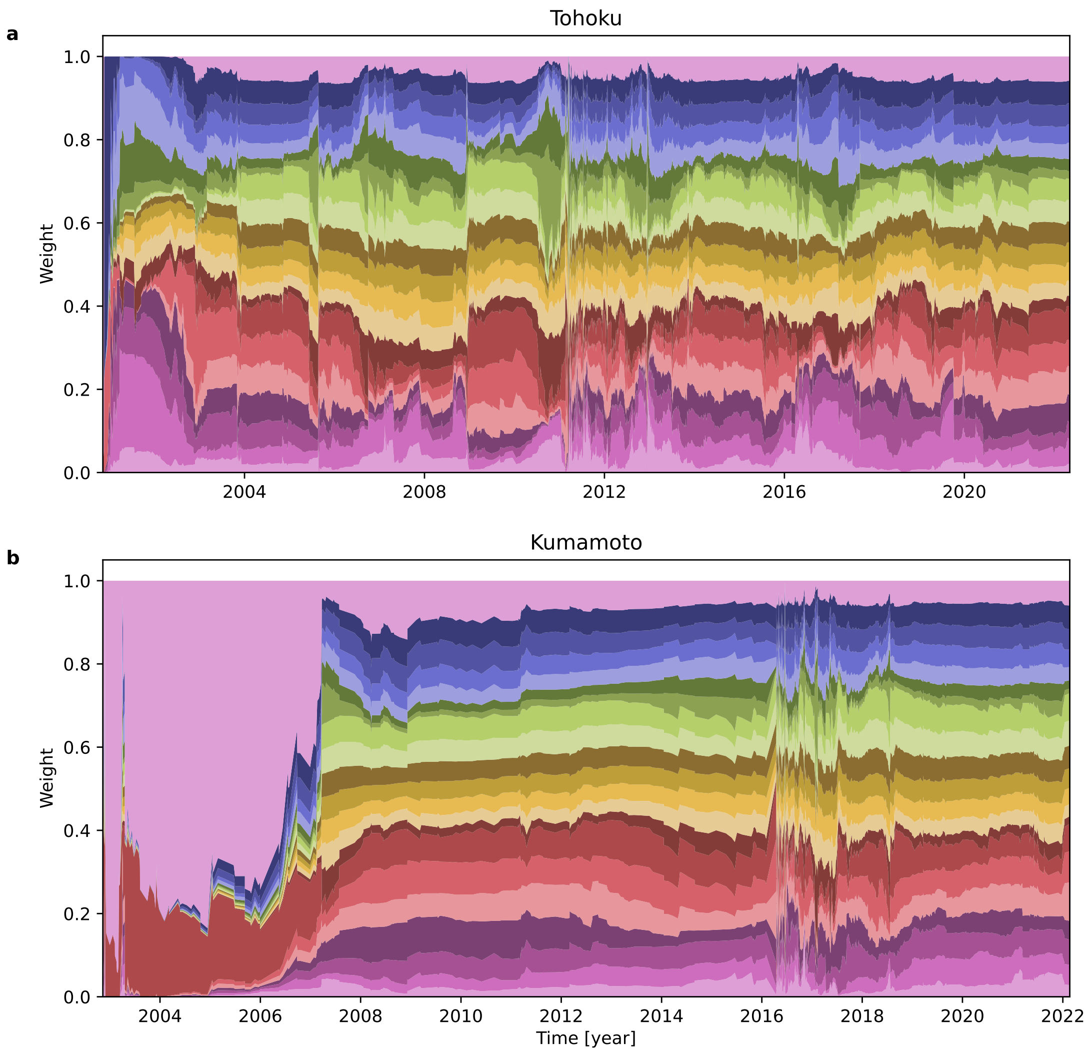

Supplementary Figure 5. Weights for applying the weighted average method. (**a**) Tohoku and (**b**) Kumamoto cases. This chart represents the weights for applying a weighted average to the results of the traditional moving average method. The stacked graph displays the weights for the results based on the settings of SMA-50, SMA-75, SMA-100, SMA-125, SMA-150, SMA-175, SMA-200, EMA-50, EMA-75, EMA-100, EMA-125, EMA-150, EMA-175, EMA-200, WMA-50, WMA-75, WMA-100, WMA-125, WMA-150, WMA-175 and WMA-200 respectively, from the bottom up. SMA, EMA and WMA represent simple moving average, exponential moving average and weighted moving average, respectively. The numbers, e.g. 50, 75, etc., represent the window width of moving average. The early period in the Kumamoto (**b**) region is a period in which the number of earthquakes was small and the b value tended to increase (Fig. 2**b**), and the weight of the long moving average window was large.

Supplementary Figure S6

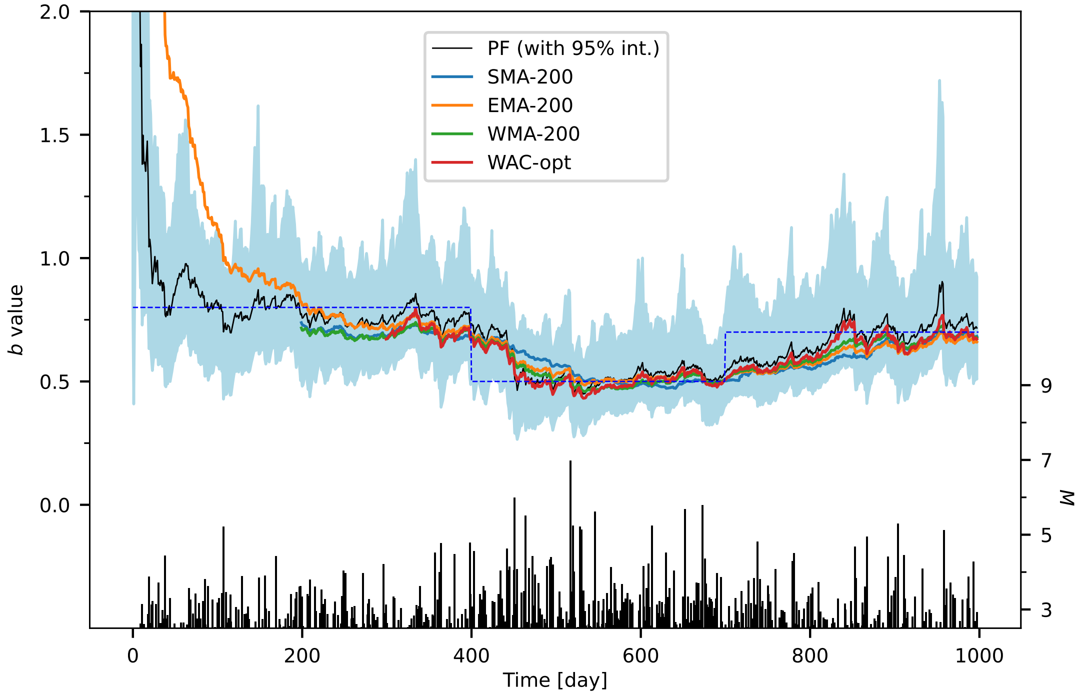

Supplementary Figure 6. Results of b value estimates for artificial data using Model 4. As described in the Method, although Model 4 is the most complex among Models 1-4, it is able to estimate b values most adaptively without optimizing any parameter. Therefore, we used Model 4 to demonstrate its behavior with test data, showcasing its performance and utility in a synthetic case. The median and 95% confidence intervals of estimated b values using particle filtering are represented by black lines and blue regions, respectively. The results of conventional moving average-based methods SMA-200, EMA-200, WMA-200 and WAC-opt are indicated with a blue, orange, green and red line, respectively. The true b value of artificial data is displayed by a blue dashed line ($0 < t \leq 400, b = 0.8$; $401 < t \leq 700, b = 0.5$; $701 < t \leq 1000, b = 0.7$). The stem plot shows magnitude generated by GR law with the true b value as a function of time. As the estimates are updated based on the data, the early estimates deviate significantly from the true values according to the initial distribution setting.

Supplementary Figure S7

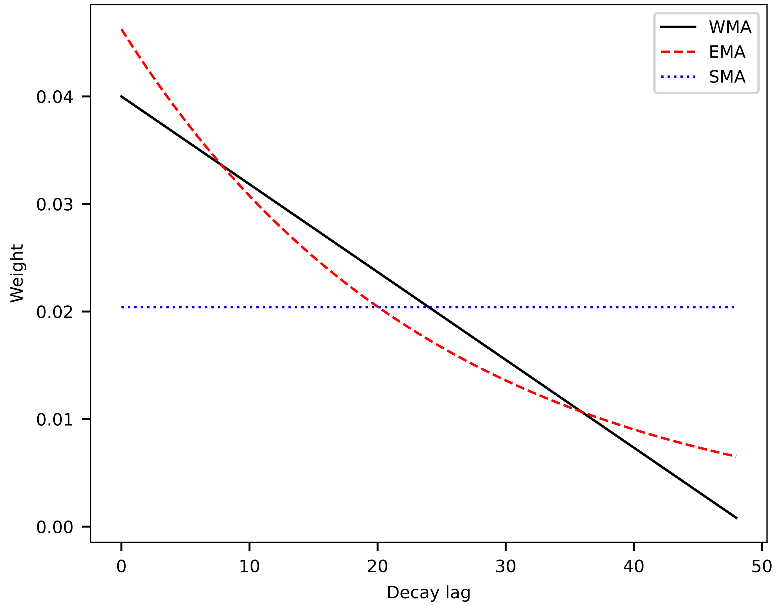

Supplementary Figure 7. Moving average kernels for one window. In the conventional moving average-based method, as illustrated, weights are applied to the values between sliding windows to compute the average. The Simple Moving Average (SMA) uses a constant weight within the window, the Exponential Moving Average (EMA) employs weights that decrease exponentially for past values, and the Weighted Moving Average (WMA) utilizes weights that decrease linearly.

Supplementary Figure S8

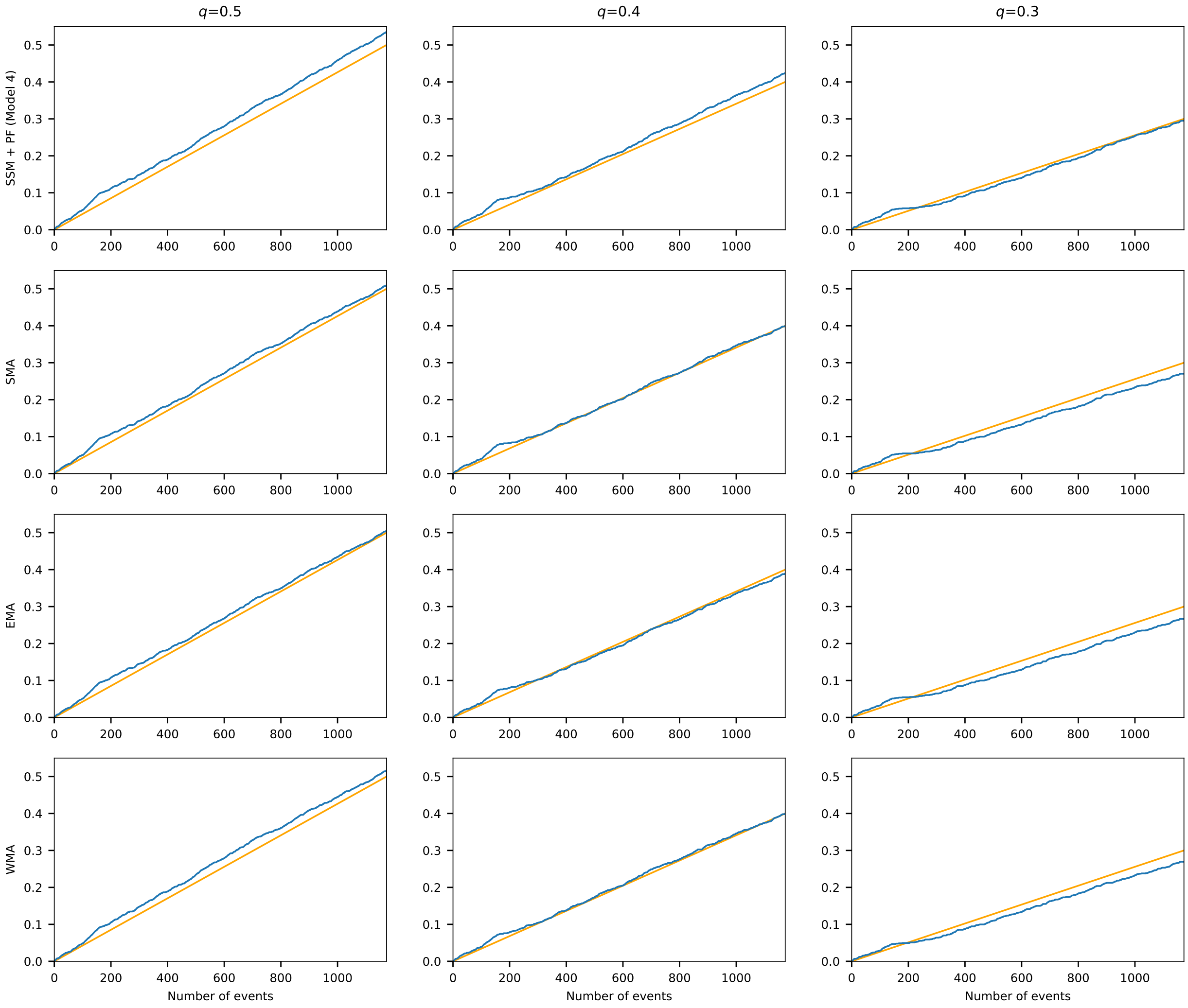

Supplementary Figure 8. The normalized number of times the cumulative count of events has surpassed the q-th percentile of the predictive distribution of magnitude by the time of the n-th earthquake, denoted as $N_{exc.}(n)$ in the Method (see Evaluation Method for Magnitude Prediction Distribution section for details). The panel displays, horizontally, the q-quantiles of the predicted distribution of magnitudes, and vertically, the results of our proposed method (SSM+PF) alongside three moving averages (SMA, EMA, WMA). The blue line represents the cumulative values of actual $N_{exc.}(n)$, while the orange line indicates theoretical values. Results in all panels are of the Tohoku dataset.

Supplementary Figure S9

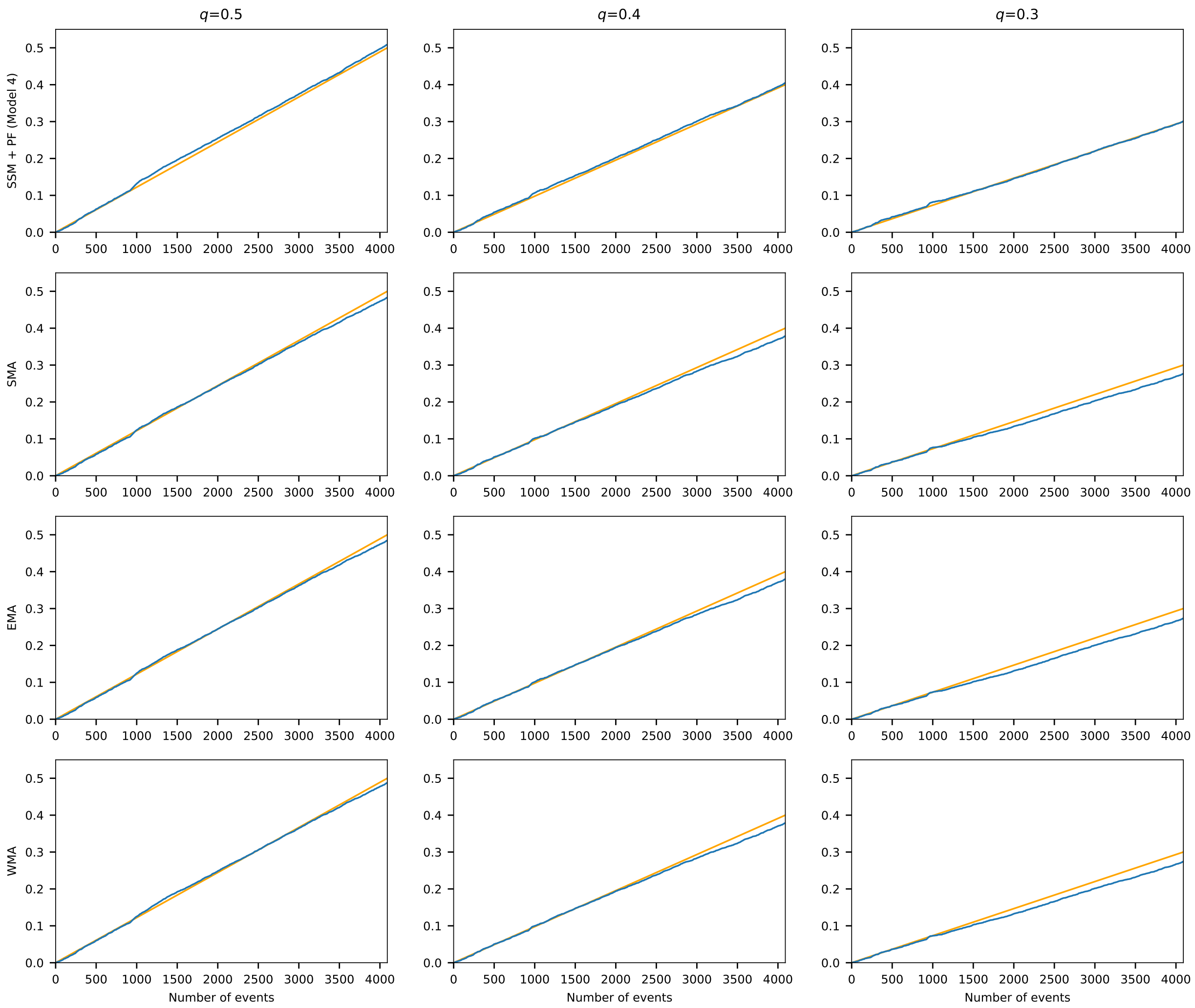

Supplementary Figure 9. The same kind of plots as Supplementary Figure S8. Results in all panels are of the Kumamoto dataset.

Supplementary Figure S10

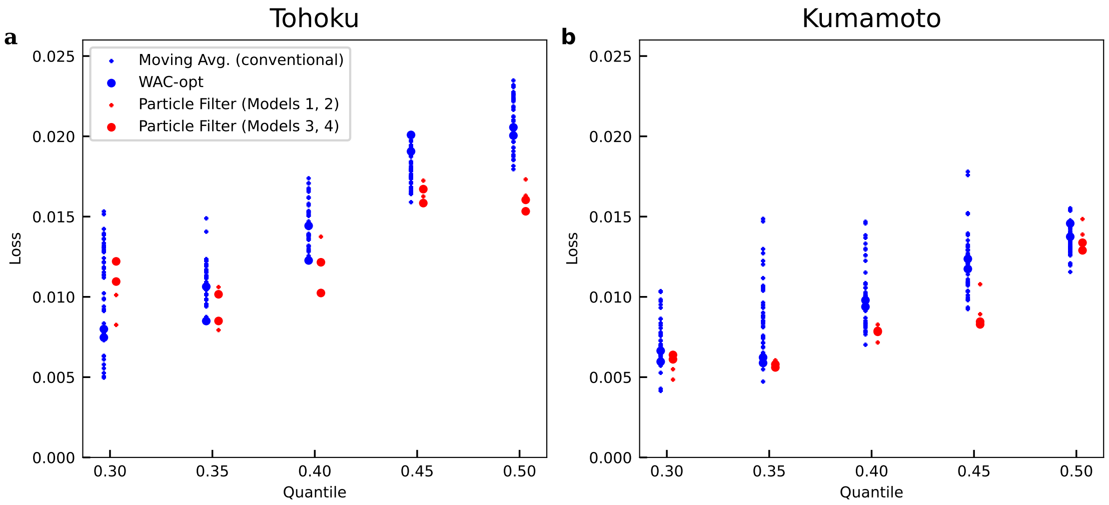

Supplementary Figure 10. The same kind of plots as Fig. 3 in main the text. The b values were estimated with conventional methods and the particle filter method with an earthquake catalog of magnitude over the magnitude completeness M_c, which was set commonly for the entire dataset. M_c were set to 2.5 and 2.0 for the analysis of the Tohoku region (**a**) and the Kumamoto region (**b**), respectively. The results of a similar analysis performed on data above M_c, calculated for each divided period, are shown in Fig. 3.

Supplementary Figure S11

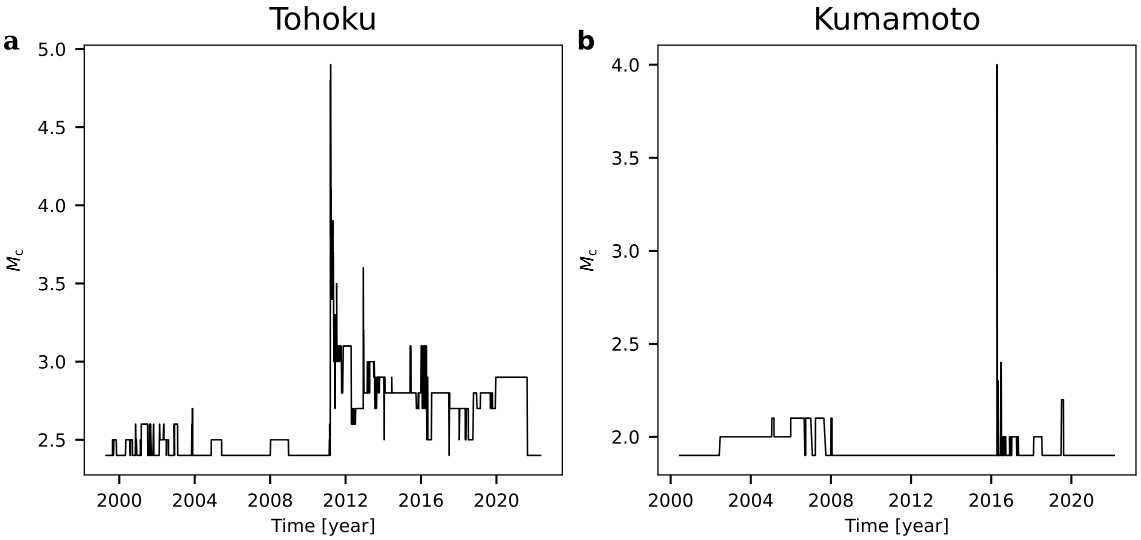

Supplementary Figure 11. Time series of the complete magnitude (M_c) calculated using the Entire-Magnitude Range (EMR) method[1], with M_c computed every 200 events. (**a**) Tohoku and (**b**) Kumamoto cases. The completeness magnitude was set to each divided period based on the time series (Tohoku: $M_c = 2.5$ before 11 March 2011, $M_c = 3.5$ between 26 March 2011 and 10 May 2011, $M_c = 3.0$ after 10 May 2011, Kumamoto: $M_c = 2.0$ before 14 April 2016 and after 20 April 2016, $M_c = 2.3$ between 14 April 2016 and 20 April 2016).

Supplementary Figure S12

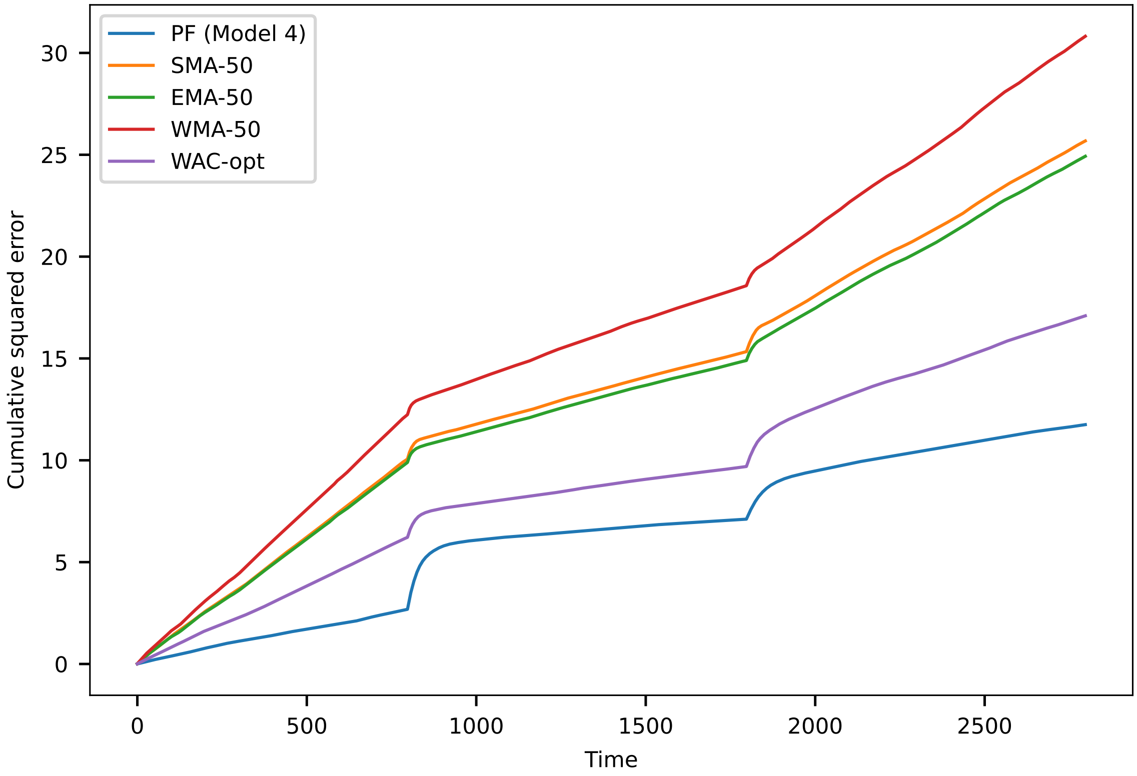

Supplementary Figure 12. Time series of the cumulative squared error between estimated b values and actual values of artificial data. Blue, orange, green, red and purple lines indicate results of different methods, particle filter, SMA-50, EMA-50, WMA-50 and WAC-opt, respectively. Each line was calculated based on the mean of 100 artificial datasets. The first 200 steps were ignored.

Supplementary Figure S13

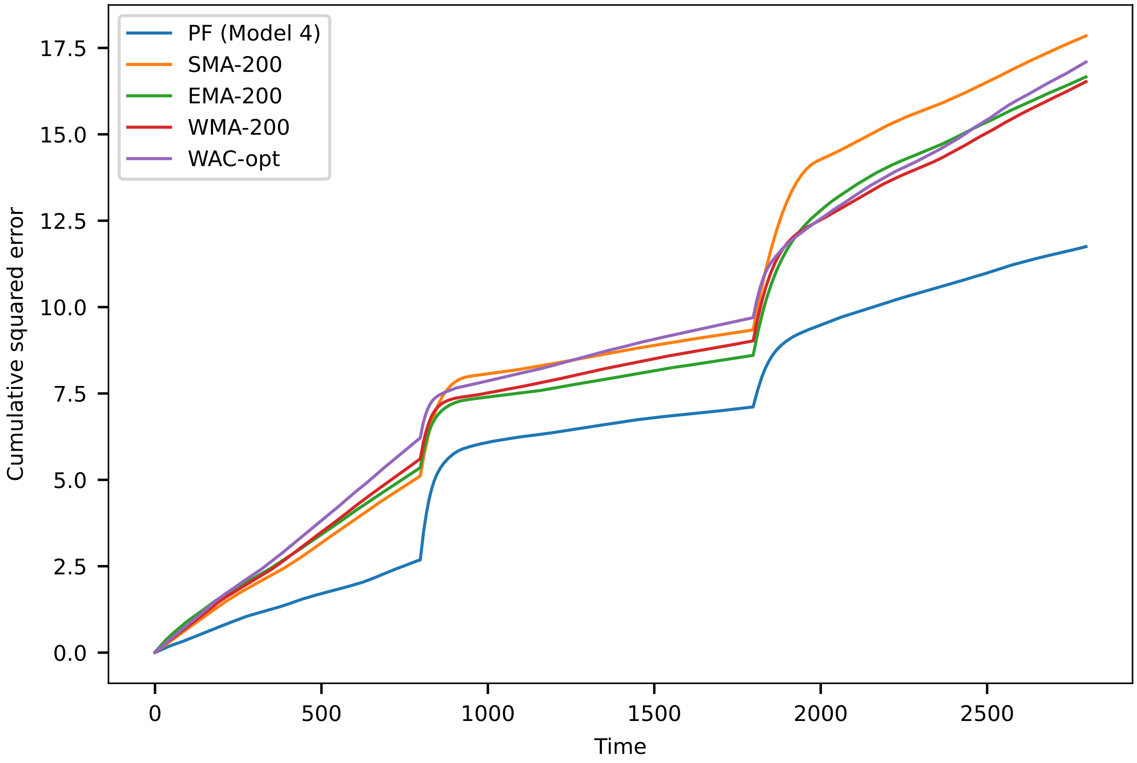

Supplementary Figure 13. The same kind of plots as Supplementary Fig. S12. Orange, green and red lines indicate the results of different methods, SMA-200, EMA-200 and WMA-200 respectively.

Supplementary Material 1

Code for analysis of the b value using a particle filter

Source codes for this study are presented in the GitHub repository, as follows:

- Repository: https://github.com/D-I-29/gr-b-pf
- Calculation environment: Python 3.10.7
- Setup environment: The environment can be set up in two ways using '`pip install -r requirements.txt`' or 'Dockerfile'. See README in the repository for details.
- Models: The method using the particle filter of Models 1 and 2 are implemented in 'particle_filter.py', while that of Models 3 and 4 are implemented in 'particle_filter_tv_.py'.

Supplementary Material 2

MSE for b value estimation

Accuracy for estimating the b value with artificial data. A total of 100 datasets of magnitude whose b values are shown in Supplementary Fig. S6 were generated, and then the b value for each dataset was estimated with conventional methods and the proposed method (Model 4). The mean and standard deviation (SD) of the Mean Squared Error (MSE) between the estimated and true values for each method are shown. Time series of cumulative squared error are displayed in Supplementary Fig. S12 and Fig. S13. Methods based on moving averages with short window lengths are quick to adapt when the b value changes, but they result in large estimation errors when the b value does not change (SMA-50, EMA-50 and WMA-50 in Supplementary Fig. S12). On the other hand, methods based on moving averages with long window lengths adapt more slowly to changes in the b value compared to those with short window lengths, but they have relatively small estimation errors when the b value remains constant(SMA-200, EMA-200 and WMA-200 in Supplementary Fig. S13). The proposed method based on particle filter demonstrates smaller estimation errors than both methods based on the moving averages of short and long window lengths.

Supplementary Table 1. Accuracy for estimating the b value with artificial data for conventional methods and the proposed method (Model 4).

	Method	MSE mean	MSE SD
0	ssm+pf_err	0.004208	0.001126
1	wac_err	0.006109	0.001336
2	sma_50_err	0.009178	0.001334
3	ema_50_err	0.008909	0.001230
4	wma_50_err	0.011014	0.001332
5	sma_75_err	0.007355	0.001388
6	ema_75_err	0.007103	0.001287
7	wma_75_err	0.008329	0.001310
8	sma_100_err	0.006632	0.001436
9	ema_100_err	0.006352	0.001341
10	wma_100_err	0.007139	0.001343
11	sma_125_err	0.006331	0.001472
12	ema_125_err	0.006011	0.001386
13	wma_125_err	0.006521	0.001374
14	sma_150_err	0.006241	0.001505
15	ema_150_err	0.005879	0.001434
16	wma_150_err	0.006180	0.001405
17	sma_175_err	0.006276	0.001513
18	ema_175_err	0.005873	0.001500
19	wma_175_err	0.005995	0.001430
20	sma_200_err	0.006380	0.001525
21	ema_200_err	0.005955	0.001604
22	wma_200_err	0.005906	0.001447

References

1. Woessner, J. & Wiemer, S. Assessing the quality of earthquake catalogues: Estimating the magnitude of completeness and its uncertainty. *Bull. Seismol. Soc. Am.* **95**, 684–698 (2005).
